# Molecular and serological characterization of occult hepatitis B among blood donors in Maputo, Mozambique

**DOI:** 10.1590/0074-02760200006

**Published:** 2020-09-23

**Authors:** Nédio Mabunda, Ana Flora Zicai, Nalia Ismael, Adolfo Vubil, Francisco Mello, Jason T Blackard, Barbara Lago, Vanessa Duarte, Milton Moraes, Lia Lewis, Ilesh Jani

**Affiliations:** 1 Instituto Nacional de Saúde, Maputo, Mozambique; 2 Fundação Oswaldo Cruz-Fiocruz, Instituto Oswaldo Cruz, Laboratório de Hepatites Virais, Rio de Janeiro, RJ, Brasil; 3 Fundação Oswaldo Cruz-Fiocruz, Instituto Oswaldo Cruz, Laboratório de Hanseníase, Rio de Janeiro, RJ, Brasil; 4 University of Cincinnati College of Medicine, Division of Digestive Diseases, United States of America

**Keywords:** occult hepatitis B, blood donors, Mozambique

## Abstract

**BACKGROUND:**

Occult hepatitis B virus (HBV) - characterized by the absence of detectable HBsAg in the presence of HBV DNA - represents a potential threat for blood safety.

**OBJECTIVES:**

This study was conducted with the aim to investigate the serological and molecular characterization of occult HBV infection (OBI) among blood donors in Mozambique.

**METHODS:**

1,502 blood donors were tested for HBsAg. All HBsAg-negative individuals were tested for HBV DNA. Antibodies against HBV core, surface and HBe antigen (anti-HBc, anti-HBs, HBeAg) were measured in HBV DNA positive individuals.

**FINDINGS:**

1435 serum samples were HBsAg negative and 16 positive for HBV DNA, 14 confirmed to have OBI, corresponding to a frequency of 0.98%. Of the 14 OBI infections identified, 13/14 (92.8%) were positive for anti-HBc, 4/14 (28.5%) for anti-HBs, and no samples were reactive for HBeAg. Of the 14 OBI cases, nine samples (64.2%) were sequenced for the S/P region. Eight samples (88.9%) belonged to genotype A1 and one (11.1%) to genotype E. One escape mutation (T123A) associated with OBI and various amino acid substitutions for genotype A1 and E were observed.

**MAIN CONCLUSIONS:**

Our results show the importance of using nucleic acid amplification test to detect occult hepatitis B infection in blood donors in Mozambique.

Significant efforts have been made to ensure safe blood for transfusion. However, several sub-Saharan African countries including Mozambique still face several challenges such as the lack of nucleic acid amplification tests (NAT).^1^ One of the main agents transmitted by blood transfusion is hepatitis B virus (HBV) that can lead to cirrhosis and hepatocellular carcinoma (HCC). Chronic HBV infection is defined by the presence of hepatitis B surface antigen (HBsAg), the main diagnostic marker for routine screening of donated blood, for at least six months in the serum.^2^ Widespread HBsAg screening using sensitive assays has substantially reduced the incidence of transfusion-transmitted HBV. However, it has been demonstrated that individuals without detectable HBsAg can transmit HBV.^3^ ^,^ ^4^ Transmission of HBV from HBsAg seronegative blood donors can occur (i) when the individual is in the window period viral exposure but before the detection of serological markers of HBV infection, or (ii) when individuals harbor HBV DNA at low levels in the absence of detectable HBsAg, a condition known as occult HBV infection (OBI).^4^

In developing countries, current HBV testing algorithms often rely on HBsAg detection alone or in combination with antibodies against viral antigens, and OBI cases are frequently undiagnosed.^5^ The presence of anti-HBc may serve as a marker of HBV infection in settings where molecular tests are unavailable. However, anti-HBc alone is not sufficient to identify OBI.^4^ ^,^ ^6^ Several mechanisms may explain the presence of HBV DNA in the absence of HBsAg, including: (i) low levels of HBV replication and HBsAg expression due to strong humoral and cellular immune response against HBsAg, (ii) mutations within the S gene that suppresses the synthesis of HBsAg or alter antigenic epitopes, (iii) the occurrence of HBsAg/anti-HBs immunocomplexes that inhibit HBsAg detection, (iv) the presence of virus reservoirs in peripheral mononuclear cells (monocytes and lymphocytes), and, (v) coinfections such as hepatitis C virus (HCV) and human immunodeficiency virus (HIV) that can interfere with HBV replication.^7^ ^,^ ^8^

Based on the HBsAg positivity in the general population, Mozambique is classified as a country with high endemicity. The prevalence of HBsAg in Mozambique among blood donors is approximately 10.6%.^9^ ^,^ ^10^ Recently, Viegas et al.,^11^ reported a higher prevalence of 12.2% in 18-24 year-olds in the capital of Maputo. Carimo et al.,^12^ reported an 8.3% frequency of OBI among patients who were anti-HBc positive. The aim of this study was to characterize serological markers (anti-HBc, anti-HBs and HBeAg), viral genotypes, and mutations associated with OBI among blood donors.

## MATERIALS AND METHODS

*Study design and population* - A cross-sectional study was performed between November 2014 and October 2015 at the Blood Bank of Hospital Central de Maputo. Blood donors that fulfilled the national blood service eligibility criteria for blood donation were included. Exclusion criteria include the donor’s general health and the risk of having a blood transfusion transmission disease as reported in full by Stokx et al.^9^ All study participants (1502 blood donors) provided written informed consent. Demographic information was obtained from all consenting blood donors using a structured questionnaire. For each participant, 9 mL of whole blood was collected into 3 mL vacutainers with K3EDTA (Becton Dickinson, Franklin Lakes, NJ, USA) and plasma sample were obtained from blood donors. The study was approved by the National Health Bioethics Committee in Mozambique (number 263/CNBS/2014).

*Serological assays* - All plasma samples were screened for HBsAg, anti-HCV, and HIV antibodies/antigens. HBV screening was performed using the Advanced Quality HBsAg ELISA Test Kit (InTec Products, INC, China), and only reactive samples were confirmed using Advanced Quality HBsAg Rapid Test (InTec Products, INC, China). HCV screening was performed using the ADVANCED Test ELISA anti-HCV (InTec Products, INC, China) and confirmed with the ADVANCED QUALITY Rapid Anti-HCV (InTec Products, INC, China). HIV screening was conducted using a qualitative enzyme immunoassay GENSCREEN PLUS HIV Ag-Ab (Bio-Rad, Marnes-l Coquette, France) and only reactive samples were re-tested using the UNIGOLD (Trinity Biotech Plc, Bray, Co. Wicklow, Ireland) rapid test. All samples were considered negative if non-reactive in the first test, and positive if reactive for both the first and second tests. Sample was considered indeterminate if the result was discordant for both tests and was not included in the next step.

*DNA quantification and detection* - HBV DNA was quantified for all HBsAg-negative blood donors in test pools of six plasma samples using the COBAS AmpliPrep/COBAS TaqMan HBV Test, v2.0 (Roche Diagnostics, Germany) with a detection limit of 20 IU/mL according to the manufacturer’s instructions. For test pools with detectable viral load, samples were individually retested using the same kit. For the individual testing 1,100 µL of plasma was used and 184 µL per sample for test pool testing. Samples with detectable HBV DNA were subsequently tested for anti-HBc and anti-HBs using the Bioelisa Kits (Biokit, Barcelona, Spain), and HBeAg levels using the Liaison Kit (DiaSorin, Sallugia, Italy) following the manufacturer’s instructions for individual blood donors.

*HBV amplification and sequencing* - HBV DNA was extracted from 200 µL of plasma using the High Pure Viral Nucleic Acid Kit (Roche Applied Science, Mannheim, Germany). The following primers were used to amplify the S and P regions: first round primers S1 (position 124-143) and 4R (position 1120-1100), and second round primers 1F (position180-203) and 4R and 1F (position180-203). The thermal cycler for the first and second cycle was programmed as follows: 30 cycles with initial denaturation at 94ºC for 1 min, 94ºC for 15 s, 56ºC for 30 s, 68ºC for 1 min and 15 s, and a final extension at 68ºC for 10 min. The amplified product was subjected to electrophoresis on a 2% agarose gel supplemented with ethidium bromide stain and visualized by UV light. High Pure Product Purification Kit (Roche, Germany) was used to purify DNA according to manufacturer’s instruction. The S/P PCR products of ~900 bases were sequenced using an automated DNA sequence 3500 Genetic Analyzer (Foster City, CA, USA) as described by Mallory et al.^13^

All laboratory activities were carried out following good laboratory practices (include routine daily practices, for example, decontamination of safety cabinets, inclusion of controls in all procedures).

*Statistical analysis* - Fisher’s exact test was used for group-to-group differences in gender, age, and donor type. Rank sum test was used to analyze HBV DNA levels. For all analyses, p-value < 0.05 was considered statistically significant.

*GenBank accession numbers* - Sequences were assigned the GenBank accession numbers MF615980-MF615989.

*Phylogenetic analysis* - Nucleotide alignments were performed with Clustal X 2.1^14^ and additional phylogenetic inference was performed using a Bayesian Markov Chain Monte Carlo (MCMC) approach as implemented in the Bayesian Evolutionary Analysis by Sampling Trees (BEAST) version 1.8.4 program^15^ with an uncorrelated log-normal relaxed molecular clock, general time-reversible model, and nucleotide site heterogeneity estimated using a gamma distribution. The MCMC analysis was run for a chain length of 100,000,000, and results were visualized to confirm adequate chain convergence with Tracer version 1.6. The effective sample size (ESS) was calculated for each parameter, and all ESS values were > 500 indicating sufficient sampling. The maximum clade credibility tree was selected from the posterior tree distribution after a 10% burn-in using Tree Annotator version 1.8.4 and visualized in FigTree version 1.4.3 as described previously.^16^ Potential recombination was evaluated using the Jumping Profile Hidden Markov Model available at http://jphmm.gobics.de/submission_hbv.

*Mutations and amino acid substitution analysis* - To identify mutations associated with occult HBV infection, a consensus sequence of HBV/A1 and HBV/E was generated from 120 and 223 GenBank references, respectively. An alignment of nucleotide sequences containing the consensus sequence and the Mozambican OBI sequences was generated and the amino acids visualized to identify substitutions occurring in the S gene ORF.

## RESULTS

A total 1,502 blood donors were screened for HBsAg, of which 1,435 (95.5%) were negative and 67 (4.5%) positive. From the 1,435 HBsAg negative samples, 16 (1.1%) had detectable HBV DNA. Two of 16 were negative for all serological markers (seronegative OBI or window period). Of the 14 (0.98%) occult HBV infections, 10 (71.4%) samples were positive for anti-HBc only, 14 (21.4%) were positive for anti-HBc and anti-HBs, and 1 (7.1%) was positive for anti-HBs only. The HBV DNA of OBI cases was significantly lower when compared to that of HBsAg-positive cases (Table I). Of the 14 OBI cases, nine samples (64.3%) could be polymerase chain reaction (PCR) amplified and sequenced, including four with an HBV viral load of < 20 IU/mL. HBV DNA levels for the remaining 5 ranged from 20 to 1326 IU/mL (Table II). No OBI case tested positive for HCV or HIV co-infection.

**TABLE I:** Characteristics of occult hepatitis B and HBsAg positive blood donors

Characteristics	HBsAg positive 67 (4.5)	Occult HBV (n = 14) 14 (0.98)	p-value
Age (years); mean (SD)	30.6 (8.6)	33.6 (10.7)	0.725
Gender			
Male; n (%)	53 (97.1)	12 (85.7)	0.248
Female; n (%)	14 (20.9)	2 (14.3)	
Donor type			
Replacement; n(%)	60 (89.6)	13 (92.9)	1
Voluntary; n(%)	7 (10.4)	1 (7.1)	
Median plasma HBV DNA, IU/mL (IQR)	1138 (149.5-7829)	23 (20-293)	< 0.001

**TABLE II:** Molecular and serological characteristics of occult HBV infection (OBI) cases in blood donors at the blood bank of Hospital Central de Maputo

Code	Serological markers			Virological characteristics		Genetic variation in HBV MHR domain	
	Anti-HBc	Anti-HBs	HBeAg	Viral load (IU/mL)	Genotypes and sub-genotypes	Substitution in MHR	Escape mutations
385	+	-	-	< 20	A (A1)	V190A, A194V, L209V, V224A	-
532	+	-	-	< 20	A (A1)	A194V	-
590	-	+	-	1014	A (A1)	A194V	T123A
800	+	-	-	28	A (A1)	A194V, S204G, L216F, F220L	-
885	+	-	-	1326	A (A1)	A159V, A194V	-
1498	+	-	-	503	E	P56L, T57I, E164G, S174N, L216*	-
1551	+	-	-	349	A (A1)	L21S, R24K/R, L49R, P70LP, F134I, V184AV, A194V, S210I/T, L216*	-
1626	+	+	-	< 20	A (A1)	W36L, A194V	-
1637	+	-	-	< 20	A (A1)	A194V	-
805	+	+	-	26	-	-	-
806	+	-	-	< 20	-	-	-
942	+	-	-	< 20	-	-	-
1629	+	+	-	< 20	-	-	-
1688	+	-	-	125	-	-	-

HBV: hepatitis B virus; MHR: major hydrophilic region. *The sign is a stop codon which means the termination of the translation process which includes an amino acid change at position 216 in the wild type virus to a stop codon.

**Fig. 1:**
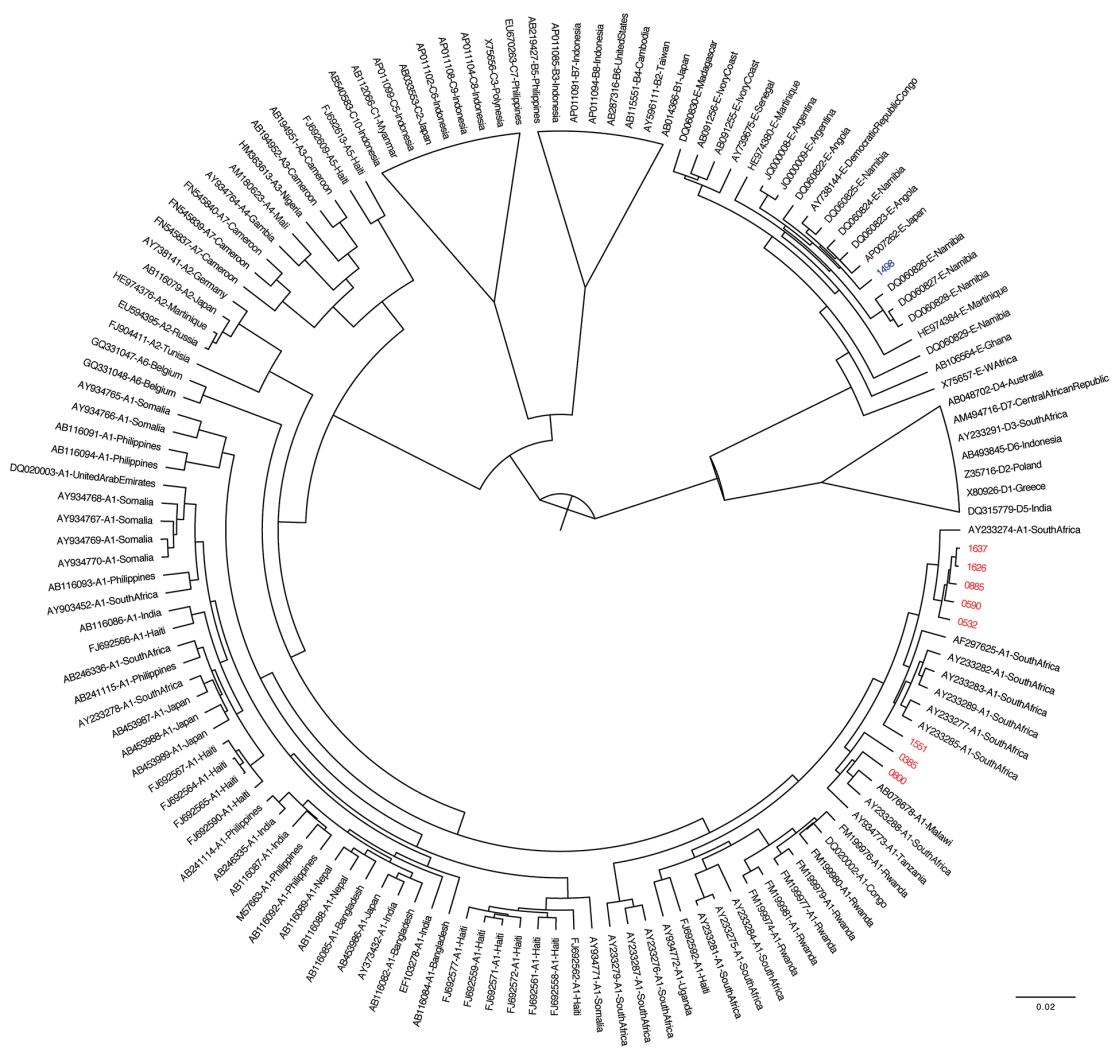
Bayesian phylogenetic analysis of partial S gene sequences from Mozambique (indicated by a 4-digit identifier) compared to reference sequences (indicated by their accession number, subgenotype, and country of origin). Hepatitis B virus (HBV) genotype A sequences (n = 8) are shown in red, while genotype E sequences (n = 1) are shown in blue.

**Fig. 2:**
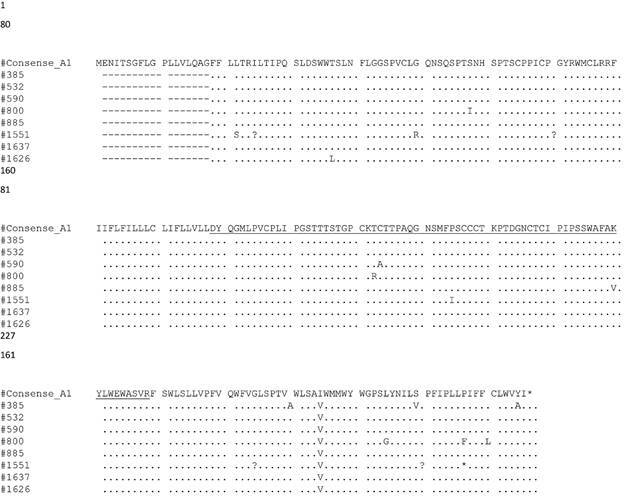
amino acid alignment of the S gene (aa 1-227) from occult hepatitis B virus (HBV)-infected individuals from Mozambique. The Major Hydrophilic Region (aa 99-169) is underlined (99-169) and the “a” determinant epitope region is represented in the box.

**Fig. 3:**
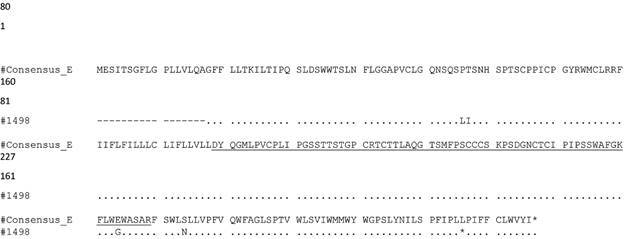
amino acid alignment of the S gene (aa 1-227) from occult hepatitis B virus (HBV)-infected individuals with genotype E from Mozambique. The Major Hydrophilic Region (aa 99-169) is underlined (99-169) and the “a” determinant epitope region is represented in the box.

Eight OBI sequences clustered with genotype A1 references, while one grouped with genotype E (Fig. 1). One escape mutation (T123A) associated with OBI in one sample was observed. Various amino acid substitutions for genotype A1 and E were observed in the major hydrophilic region (MHR). In addition, polymorphism I195V within the S gene for all the samples was also detected in OBI individuals belonging to genotype A1 (Table II, Figs 2-3).

## DISCUSSION

The present study is the first to identify and characterize OBI cases among blood donors in Mozambique, and highlights the presence of HBV DNA despite undetectable HBsAg and the absence of other serological markers. Fourteen OBI cases were identified among 1,435 HBsAg-negative blood donors an overall prevalence of 0.98%.

Studies conducted in Colombia and Nigeria found a prevalence of 1.98% and 17% among blood donors, respectively. Both studies tested for HBV DNA amongst all HBsAg-negative and anti-HBc positive samples.^17^ ^,^ ^18^ One the other hand, study that included blood donors from South Africa, Egypt, The Mediterranean, Northern and Central Europe, Southeast Asia and Oceania showed OBI rates ranging from 1:3,900 to 1:59,000.^19^ Although OBI data in sub-Saharan African is limited, other studies in Botswana and South Africa have identified OBI in 6.6% to 15% in HIV-infected patients.^20^ ^,^ ^21^ In this study, no hepatitis C or HIV co-infections were identified.

Recently, a study conducted in HIV patients naive to antiretroviral therapy in Mozambique reported an OBI prevalence of 8.2%.^12^ The difference in the prevalence reported in our study and that of Carimo et al. may be attributed to (i) differences in the study population group (HIV patients are at higher risk of OBI due to immunodeficiency), (ii) the inclusion criteria utilized by Carimo et al. that may lead to over or underestimation of OBI cases. With this criteria, only patients with anti-HBc alone were submitted to PCR assay and a portion of HBsAg gene was amplified and (iii) the pooling method used to determine HBV DNA might explain the low frequency of OBI observed in the current study.

Significantly lower levels of HBV DNA were detected in OBI samples, with the exception of two samples (BSM 885 and 590). This low level HBV DNA is in accordance with previous studies, indicating strong suppression by the immune response together with histological derangements occurring during acute or chronic HBV infection.^22^ In contrast, the presence of high HBV DNA in two samples may be associated with recent infection increasing the risk of infectivity in the transfused blood. Nevertheless, in agreement with other studies, other factors not observed in this study such as host factors and epigenetic modifications may account for the occult HBV phenotype observed in these individuals.^23^ In fact, *in vitro* studies have demonstrated that OBI strains are competent for replication.^24^

Of the 14 identified OBI cases, nine were sequenced of which eight belonged to sub-genotype A1. Seven sequences were similar to South African sequences, one to a Tanzania sequence, and one to genotype E (Fig. 1). These findings may be explained by the geographic proximity of Mozambique to South Africa and Tanzania. The present study revealed no difference in HBV genotypes observed between the occult HBV population and the HBV population co-infected with HIV previously observed in sub-Saharan countries where sub-genotype A1 predominates. Indeed, it has been proposed that genotype A originated in Africa with sub-genotype A1 from sub-Saharan Africa and India.^25^ Genotype E is most prevalent in West Africa but have been reported sporadically in other African countries.^26^ The introduction of genotype E is probably due to increased population migration occurring in recent years between Mozambique and West African countries. However, full genome sequencing and further phylogenetic analyses will be necessary to validate this hypothesis.

Understanding the immunologic and molecular characteristics that play a role in OBI cases determination is important and must be investigated further. Various studies propose the alteration of HBsAg antigenicity, inhibiting anti-HBs production associated with escape mutants in OBI cases within the PreS/S region. Changes in the epitope, such as a single mutation in the “a” determinant (amino acid 124-147), inhibit HBsAg secretion. On the other hand, amino acid substitutions in the reverse transcriptase (RT) domain can also result in low levels of HBV DNA and HBsAg synthesis influencing occult infection.^27^ In our study, amino acid substitutions were found within the MHR region in all samples. Substitutions V190 and F134I in genotype A samples have been reported in some studies, however their contribution to occult hepatitis has not yet been described.^18^ ^,^ ^28^ Further studies are needed to determine effects of these and other variation we found in this study on HBsAg and HBV. In particular, T123A escape mutation located in the MHR of the S gene was distinctly present in one patient (sample BSM590). This finding is in concordance with several studies reporting the occurrence of T123A mutation in OBI individuals, changing its immunogenicity and making HBsAg unrecognizable by available commercial kits. The ability of different commercial tests to detect HBsAg in sample with escape mutations varies, reason why testing the same sample with different commercial tests is recommended.^27^ ^,^ ^29^

Furthermore, the A194V polymorphism observed among genotype A1 sequences in this study may represent a molecular signature for the HBV Mozambican infected population; however, further studies must be conducted to evaluate this hypothesis.

With regards to additional analysis for sample BSM1498 belonging to genotype E, amino acid substitution E164G was observed within the MHR. Interestingly, amino acid substitution E164G was also observed in a study conducted among Nigerian blood donor samples.^18^

In the present study, individuals positive for anti-HBc, anti-HBs and HBV DNA were observed, representing a viral persistency with low viral load as observed in other studies as well.^18^ ^,^ ^20^ ^,^ ^21^ A plausible explanation for this may be related to poor neutralization by the anti-HBs antibody that leads to the loss of recognition allowing the mutant virus to escape to neutralization in the presence of the protective antibody. Notably, HBeAg detection was not observed in the present study suggesting the absence of wild type variants previously described in HBV HBeAg positive individuals. Most individuals positive for anti-HBc only had a viral load below the detection limit in concordance with the theory by Prati et al.^30^ which states that anti-HBc positivity alone contributes to low viral replication in OBI individuals.

Limitations of the current study include the lower limit of detection of the HBV DNA assay (20 UI/mL). Second, only participants with the eligibility for blood donation according to Stokx et al.^15^ were included in this study fact that may have influenced the with the overall low frequency of OBI infection detected in our study. Nevertheless, the detection of 16 out of 1,435 samples screened indicates that molecular approaches should utilized for routine blood screening to avoid HBV transmission.

*In conclusion* - This study is the first to demonstrate that OBI is prevalent among healthy asymptomatic blood donors in Mozambique. The presence of 0.98% detectable HBV DNA among negative HBsAg individuals suggests that asymptomatic blood donors with HBV infection will not be identified when screened with commonly available serological tests at the blood banks in Mozambique, increasing the risk of HBV transmission. The introduction of routine molecular screening tests for blood donation may prevent the transmission of HBV in the window period and occult hepatitis B. Mutations detected within the S gene and MHR region may have accounted for the occult nature of HBV infection in these blood donors observed in Mozambique.
